# A Machine Learning Model Based on MRI Radiomics to Predict Response to Chemoradiation Among Patients with Rectal Cancer

**DOI:** 10.3390/life14121530

**Published:** 2024-11-22

**Authors:** Filippo Crimì, Carlo D’Alessandro, Chiara Zanon, Francesco Celotto, Christian Salvatore, Matteo Interlenghi, Isabella Castiglioni, Emilio Quaia, Salvatore Pucciarelli, Gaya Spolverato

**Affiliations:** 1 Institute of Radiology, Department of Medicine-DIMED, University of Padova, 35128 Padova, Italy; filippo.crimi@unipd.it (F.C.); carlo.dalessandro@studenti.unipd.it (C.D.); zanon.chiara.9@gmail.com (C.Z.); emilio.quaia@unipd.it (E.Q.); 2 Third Surgical Clinic, Department of Surgical, Oncological and Gastroenterological Sciences (DiSCOG), University of Padova, 35128 Padova, Italy; celotto.francesco92@gmail.com (F.C.); puc@unipd.it (S.P.); 3 DeepTrace Technologies S.R.L., Via Conservatorio 17, 20122 Milano, Italy; christian.salvatore@iusspavia.it (C.S.); interlenghi@deeptracetech.com (M.I.); 4 Department of Science, Technology and Society, University School for Advanced Studies IUSS Pavia, 27100 Pavia, Italy; 5 Dipartimento di Fisica Giuseppe Occhialini, Università degli Studi di Milano Bicocca, Piazza della Scienza 3, 20126 Milano, Italy; isabella.castiglioni@unimib.it

**Keywords:** MRI, radiomics, rectal cancer, neoadjuvant therapy, machine learning

## Abstract

Background: With rectum-sparing protocols becoming more common for rectal cancer treatment, this study aimed to predict the pathological complete response (pCR) to preoperative chemoradiotherapy (pCRT) in rectal cancer patients using pre-treatment MRI and a radiomics-based machine learning approach. Methods: We divided MRI-data from 102 patients into a training cohort (*n* = 72) and a validation cohort (*n* = 30). In the training cohort, 52 patients were classified as non-responders and 20 as pCR based on histological results from total mesorectal excision. Results: We trained various machine learning models using radiomic features to capture disease heterogeneity between responders and non-responders. The best-performing model achieved a receiver operating characteristic area under the curve (ROC-AUC) of 73% and an accuracy of 70%, with a sensitivity of 78% and a positive predictive value (PPV) of 80%. In the validation cohort, the model showed a sensitivity of 81%, specificity of 75%, and accuracy of 80%. Conclusions: These results highlight the potential of radiomics and machine learning in predicting treatment response and support the integration of advanced imaging and computational methods for personalized rectal cancer management.

## 1. Introduction

Colorectal cancer is the third most common cancer in males and the second most common in females worldwide, accounting for 10% of the total cancer burden [1], and rectal cancer accounts for approximately one-third of these cases [2].

Rectal cancer remains a major challenge in oncology, prompting continuous exploration of innovative management strategies [3]. A notable paradigm shift in this landscape is represented by rectal-sparing approaches, particularly for patients achieving a complete or major response following neoadjuvant therapy. This evolving paradigm signifies a potential departure from traditional radical surgical interventions and holds the promise of preserving rectal function and quality of life. 

The adoption of rectal-sparing approaches remains contentious and necessitates a nuanced understanding of their role in the comprehensive management of rectal cancer [4].

The emergence of neoadjuvant therapy, including radiotherapy and chemotherapy, has significantly altered the treatment landscape for rectal cancer [5]. Patients who exhibit a robust response to these interventions, achieving a complete or major response, present a unique cohort where sparing the rectum from radical surgery becomes a plausible consideration. The prospect of rectal sparing introduces a delicate balance between maximizing oncological outcomes and minimizing the potential morbidity associated with extensive surgical procedures.

Despite the potential benefits, the application of rectal-sparing approaches is hampered by controversies surrounding patient selection. Identifying individuals most amenable to rectal sparing is pivotal in realizing the full potential of this paradigm shift [6]. Improved precision in patient selection requires a comprehensive understanding of factors influencing treatment response and the ability to predict outcomes accurately.

Magnetic resonance imaging (MRI) is the best diagnostic tool for local staging and restaging due to its excellent image resolution; it also plays an essential role in the evaluation of treatment response, surveillance, and in the detection of local recurrence and surgery [7].

In particular, it plays a pivotal role in the local staging of the disease both before and after pCRT in order to achieve comprehensive information regarding tumor (T) and node (N) staging, extramural venous invasion (EMVI), and circumferential resection margin (CRM), which can guide the decision about the most appropriate clinical and therapeutic approach to undertake [8].

MR image assessment has recognized limitations that are pushing the research towards the identification and validation of innovative strategies to further increase the value of radiologic study; that is the reason why a post-processing quantitative technique known as radiomics appears particularly interesting and promising for the goal [9]. Trying to quantify what is visually assessed in radiological imaging is a challenging task, and radiologists have traditionally provided few qualitative and semi-quantitative data and information in their structured reports [10]. Radiomics aims to translate medical images into a large number of quantitative features, namely morphological and texture features that express the heterogeneity of local disease, defined as surrogate biomarkers of disease, which may reveal a deeper level of detail than that which is accessible to the unaided human eye so as to quantify tumor phenotypes, which could aid in clinical decision-making [8].

Against this backdrop, our study aims to contribute to the ongoing discourse on rectal cancer management by integrating advanced imaging technologies, such as magnetic resonance imaging (MRI), with sophisticated computational methodologies taking advantage of machine learning methods. The exploration of radiomic-based machine learning models aligns with the broader objective of discerning subtle patterns indicative of treatment response. By harnessing the potential of radiomic features, we seek to refine patient stratification, facilitating a more informed and personalized approach to rectal cancer treatment planning [11].

The overarching goal is to address the controversies surrounding rectal-sparing approaches [12] by providing, through MRI radiomics analysis, insights into the identification of patients who stand to benefit the most from such strategies. Through a systematic and comprehensive analysis, we aim to contribute valuable knowledge that could address clinical decision-making and potentially reshape the management landscape for rectal cancer. The role of rectal sparing in enhancing patient outcomes, reducing morbidity, and improving overall quality of life underscores the urgency of advancing our understanding and refining patient selection criteria in this evolving era of rectal cancer management.

Hence, the aim of the present study was to develop, train, and assess the accuracy of a radiomic model based on staging axial T2-weighted MR images for rectal cancer in the prediction of the complete response of the tumor to chemoradiotherapy.

## 2. Materials and Methods

### 2.1. Patients

The present research was a substudy of a large prospective study approved by the local Ethics Committee. The study followed the principles of the Declaration of Helsinki, and all patients gave their written, informed consent. The study was reported in line with the STARD (Standards for the Reporting of Diagnostic accuracy studies) criteria.

**Figure 1:**
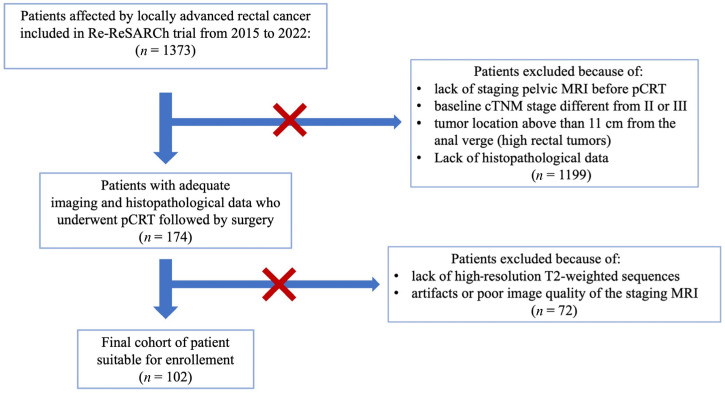
Flowchart of the study population accrual.

Figure 1 summarizes the study population accrual according to enhancing the quality and transparency of health research guidelines [13]. Starting from an initial pool of 1373 patients, we included only patients with a confirmed diagnosis of locally advanced rectal cancer who underwent pCRT followed by curative intent surgery (total mesorectal excision) at our center between 1 January 2016 and 31 December 2022. Inclusion criteria were as follows: rectal cancer with baseline cTNM stage of II or III; tumor location less than 11 cm from the anal verge (low-middle rectal tumors); patients undergone neoadjuvant therapy (5-fluorouracil or capecitabine-based chemotherapy and fractioned radiotherapy with a total dose of 50.4 Gy); and availability of the results of histopathological examination for T and N staging. Thus, the study cohort included 174 patients. Exclusion criteria were the following: lack of staging pelvic MRI before pCRT, lack of high-resolution T2-weighted sequences and artifacts, or poor image quality of the staging MRI. These criteria are critical to the external validation of the radiomics model as they ensure consistent and reliable selection of the study population. Finally, the study population included 102 patients (median age: 64 years; interquartile range: 55–72 years), with 58 (56.8%) men and 44 (43.2%) women.

### 2.2. Imaging Protocol

The MRI scans were performed using a 1.5T MRI scanner (MAGNETOM Avanto; Siemens Healthineers, Erlangen, Germany). Before examination, patients were asked to perform a rectal enema, and scopolamine butylbromide (Buscopan [Sanofi-Aventis, Paris, France]) was injected as an antispasmodic drug if there were no contraindications. Patients assumed the supine position and endorectal filling with ultrasound gel was performed. The MRI examinations were performed on the basis of the protocol suggested by the European Society of Gastrointestinal and Abdominal Radiology (ESGAR) guidelines [14], including T2-weighted high-resolution turbo spin echo sequences in sagittal, oblique axial (perpendicular to the long axis of the tumor), and oblique coronal (parallel to the long axis of the tumor) with a slice thickness of 3.5 mm, repetition time [TR] between 3790 ms and 5354 ms, and a 119 ms echo time [TE]; diffusion-weighted imaging (DWI) oblique axial sequences (slice thickness 3.5 mm; TR of 3200 ms and TE of 79 ms; b 50, 500 and 1000 s/mm^2^; apparent diffusion coefficient (ADC) maps; and sagittal, oblique coronal, and oblique axial T1-weighted volumetric interpolated breath-hold examination (VIBE) sequences (slice thickness 3.5 mm; TR of 5.27 ms and TE of 1.87 ms; flip angle of 12°) 60 s after intravenous administration of a gadolinium-based contrast agent (gadobutrol [Gadovist, Schering AG, Berlin, Germany] 0.1 mL/kg up to a maximum of 16 mL, flow rate 2.0–2.5 mL/s), followed by a 20 mL saline flush.

### 2.3. Image Sets

For the radiomics analysis, we used the high-resolution T2-weighted images in oblique axial plane.

We collected a total of 72 subjects’ MR images as a training cohort, comprising 52 patients (72.2%) classified as “Not responding” to pCRT (i.e., residual disease) and 20 patients (27.8%) classified as “complete response (pCR)” based on the histological diagnosis from definitive surgery (reference standard). We used these images for training, cross-validation, and internal testing of three distinct machine learning models.

Separately, we used another group of 30 patients’ MR images as an external validation cohort. Of these, 26 patients were classified as not responding, and 4 patients were classified as pCR by the reference standard.

### 2.4. Radiomic-Based Machine Learning Modeling

Radiomic analysis followed the guidelines established by the International Biomarker Standardization Initiative (IBSI) [15]. The Trace4Research^TM^ radiomic platform (DeepTrace Technologies s.r.l., Milano, Italy) was employed for its comprehensive and fully automated IBSI-compliant workflow [16]. This encompassed the segmentation of the volume of interest (VOI), preprocessing of image intensities within the segmented VOI, computation of radiomic features, and subsequent application of these features to train, validate, and internally test machine learning classifiers for the binary classification task (“Not responding” vs. “pCR”).

**Figure 2:**
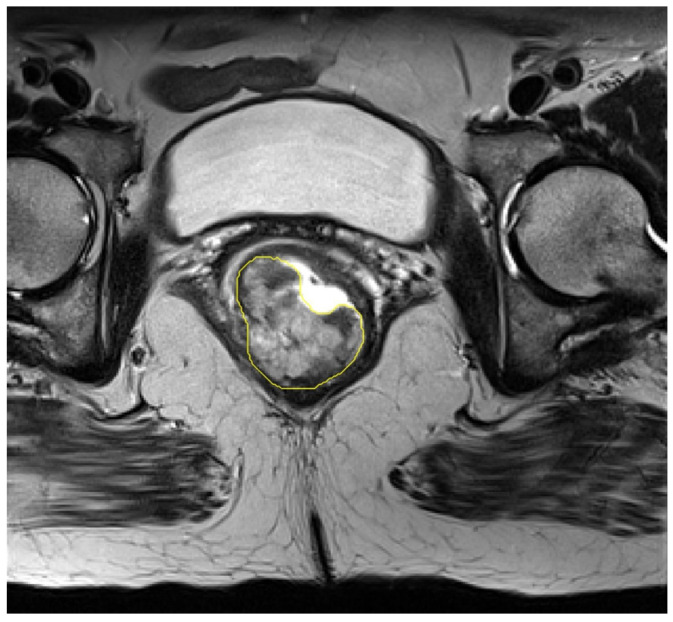
MRI T2-weighted axial oblique scan with endorectal filling of rectal cancer, manually segmented VOI (yellow line).

Specifically, the workflow included the following steps:

1. Manual Segmentation: the VOI segmentation on the T2-wighted images was meticulously performed manually, slice by slice, by two expert operators (5 years and 10 years of experience in abdominal radiology and MRI rectal cancer staging) in consensus, blinded to clinical and histopathological data, utilizing the Trace4Research segmentation tool (Figure 2).

2. Image Intensity Preprocessing: Intensity preprocessing within the segmented VOI involved resampling to isotropic voxel spacing. A downsampling scheme based on the greatest dimension of the VOI was employed, capping the mask size at 10 million voxels for texture features and 1 million for other features.

3. Radiomics Feature Computation: Radiomic features from the segmented VOI spanned various families, such as morphology, intensity-based statistics, intensity histogram, gray-level co-occurrence matrix (GLCM), gray-level run length matrix (GLRLM), gray-level size zone matrix (GLSZM), neighborhood gray tone difference matrix (NGTDM), and neighboring gray level dependence matrix (NGLDM). The computation adhered to IBSI guidelines, with texture features computed after intensity discretization (64 bins).

4. Feature Selection: Features with low variance (threshold = 0.1) were removed, and features with low mutual-information with the class label were eliminated through mutual-information analysis (selecting features with mutual information > 0.28). The selected informative and nonredundant radiomic features were reported according to IBSI standards.

5. Machine Learning Classification

5.1. Machine learning algorithms: three machine learning models were trained, validated, and tested for the binary classification task using histological diagnosis as the reference standard.

- The first model was based on the random forest (RF) algorithm combined with the Gini index, principal components analysis (PCA), and the Fisher discriminant ratio (FDR) with mean-vote rule.
- The second model was based on a support vector machine (SVM) algorithm combined with PCA and FDR with a mean-vote rule.
- The third model used a k-nearest neighbor (kNN) algorithm combined with PCA and FDR with a mean-vote rule.

These machine learning algorithms were chosen because they are well-established state-of-the-art algorithms characterized by high performance in both accuracy and training time [17].

The three trained models included three ensembles of RF, SVM, and kNN classifiers, respectively.

5.2. Dataset unbalance: oversampling for the minority class (“pCR”) was applied using the adaptive synthetic sampling method (ADASYN).

5.3 Training method: models were trained, validated, and tested using a nested 10-fold cross-validation method, ensuring high levels of robustness since it randomly splits data into subsets, separating data used for training and validation from data used for performance evaluation.

5.4 Model performance evaluation: For each of the three models, training, validation, and internal testing performances were calculated as means in terms of area under the receiver operating characteristic curve (ROC-AUC), accuracy, sensitivity, specificity, positive predictive value (PPV), and negative predictive value (NPV), with their respective 95% confidence intervals (CI). Internal testing performances were also computed as majority-vote consensus among the classifiers. The model with the highest internal testing mean ROC-AUC was chosen as the optimal classification model since a higher ROC-AUC value indicates a higher predictive ability of the model for the classification task.

### 2.5. Statistical Analysis

Statistical analyses were conducted using the embedded tools of the Trace4Research platform. Training, validation, and internal testing performance were reported with their 95% CI, and *p*-values for one-sided Wilcoxon signed rank tests were performed to assess statistical significance with respect to chance/random classification, using as significant levels 0.05 (*) and 0.005 (**).

To characterize the distribution of relevant features in the “Not responding” and “pCR” classes, medians with 95% confidence intervals were calculated, and violin and box plots were generated for visual representation.

A non-parametric univariate Wilcoxon rank-sum test (Mann–Whitney U test) was performed for each relevant radiomic predictor to assess its significance in discriminating between “Not responding” and “pCR” classes. To address multiple comparisons, *p*-values were adjusted using the Bonferroni method, and significance levels were set at 0.05 (*) and 0.005 (**).

## 3. Results

**Figure 3:**
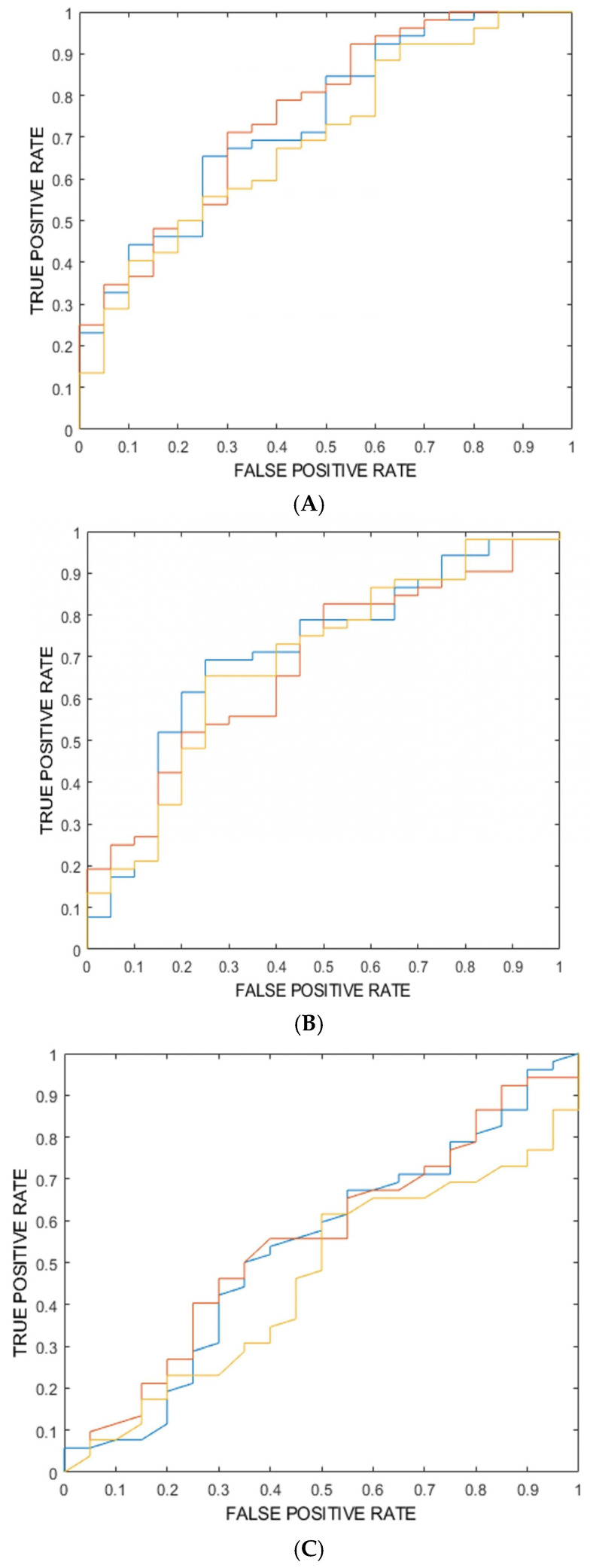
(**A**) ROC curve for the model consisting of 3 ensembles of random forest classifiers (from internal testing). (**B**) ROC curve for the model consisting of 3 ensembles of support vector machine classifiers (from internal testing). (**C**) ROC curve for the model consisting of 3 ensembles of k-nearest neighbor classifiers (from internal testing).

From each segmented VOI of each image considered in this study, the radiomic platform computed a total of 802 IBSI-compliant radiomic features and selected those that resulted in non-redundant and informative ad statistical analysis (for a total of six features). For the classification task of interest (52 images from class “Not responding” vs. 20 images from class “pCR”), these features were used for training, cross-validation, and internal testing (nested 10-fold cross validation) of three different models of machine learning classifiers considered in this work. Each trained classifier selected the most relevant features for the classification task based on its proper algorithm (i.e., Gini index for the RF model and PCA and FDR for the SVM and kNN models). Table 1A–C show ROC-AUC, accuracy, sensitivity, specificity, PPV, and NPV as obtained from the training, validation, and internal testing of the three models consisting of three ensembles of machine learning classifiers. Furthermore, for each model, ROC curves for the three ensembles are plotted in Figure 3A–C.

Based on ROC-AUC, the model of random forest classifiers resulted to be the best model for the task of interest (52 images from class “Not responding” vs. 20 images from class “pCR”).

In the external validation cohort of 30 patients, the test of the best model showed a sensitivity of 80.8% (21/26), a specificity of 75.0% (3/4), and an accuracy of 80.0% (24/30).

**Figure 4:**
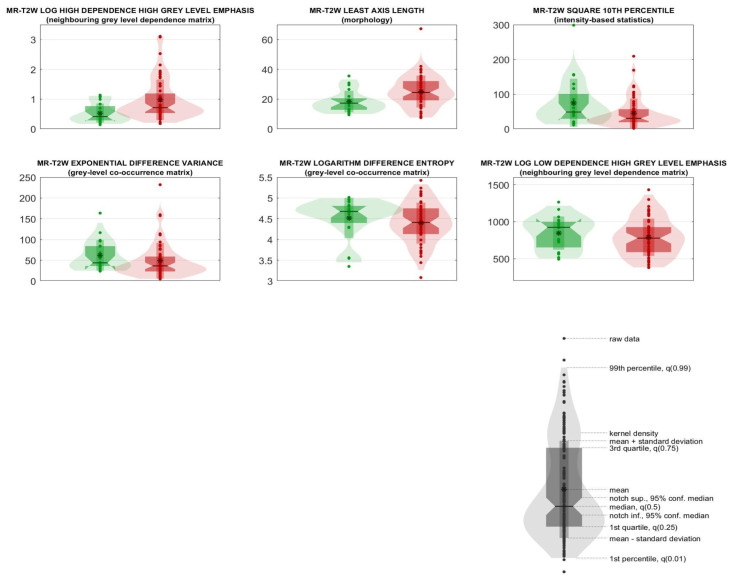
Ensemble of random forest. Violin and box plots of the radiomic predictors ranked from 1 to 6, “Not responding” and “pCR” classes are reported in red and green, respectively.

The six radiomic predictors are shown in Table 2 together with their IBSI feature family and feature nomenclature. Predictors are ranked according to their statistical significance and their frequencies among the most relevant ones in the ensemble of random forest classifiers. Median values of each feature, 95% CIs, and results from univariate statistical rank-sum tests are also reported with adjusted *p*-values. The violin plot and boxplot of the radiomic predictors are shown in Figure 4.

## 4. Discussion

The response to neoadjuvant chemoradiation therapy guides the course of treatment and the decision about the possible choice of surgery in patients with rectal cancer. Predicting such response can be critical, especially for those patients with low rectal cancer in whom radical surgery results in a major alteration in quality of life [18].

In this study, the radiomics-based approach on MR images acquired before neoadjuvant therapy showed good accuracy with the best model for the identification of the pCR after pCRT in rectal cancer patients (i.e., 70% and 80% for internal and external testing, respectively).

High sensitivity and positive predictive value (PPV) (on the order of 80% both in internal and external testing) warrants good accuracy in detecting those patients at risk to not respond to pCRT for their prompt addressing to traditional surgery.

Regarding the most significant radiomic biomarkers and their explainability, MR-T2W_LoG_high dependence high gray level emphasis is a measure describing the texture of tissue within the lesion capturing clusters with high signal intensities and showed higher values in non-responding lesions with respect to responding lesions (7120.79 [5690.59–8550.98] vs. 4117.66 [2431.26–5804.06]). MR-T2W_least axis length is a morphological measure describing the length of least axis of the lesion volume and showed higher values in non-responding lesions with respect to responding lesions (24.48 [21.68–27.28] vs. 17.27 [14.56–19.99]). Lesions with a more compacted and bigger shape and characterized by more contrasted tissue clusters seem to be more at risk of not responding to pCRT.

This analysis on the staging MRI opens new scenarios in tailoring the best therapeutic approach for patients affected by rectal cancer. Indeed, the possibility of identifying the patients that could respond better to neoadjuvant treatment since the initial staging would allow to choose more accurately those who should be addressed to rectal-sparing approaches, such as “watch and wait” or local excision instead of traditional surgery.

In the literature, there are many papers dealing with MRI radiomic models for pCR prediction after neoadjuvant therapy, showing how the topic is interesting in light of the possible use of rectum-sparing approaches that have been shown to reduce compliances and co-morbidities in rectal cancer patients with a clinical major/complete response to chemoradiation without a significant change in the outcome compared to traditional surgery.

Among the most recent studies, few dealt only with radiomic models based on T2-weighted images, and one dealt exclusively with pre-treatment T2-weighted sequences. 

In particular, Yardimci A.H. et al. [19] demonstrated that a model based on radiomic features extracted from pre-treatment sagittal T2-weighted MR images had 81% accuracy in predicting pCR after pCRT in their validation cohort. This result, obtained in a larger cohort of patients (*n* = 23 with pCR), matches the accuracy of our model. Similar to our study, Yardimci A.H. et al. used radiomic features of T2-weighted images for their model. However, the difference is that the model we presented used high-resolution T2-weighted images in an axial oblique projection, which provides better tumor definition than the sagittal projection. Validation was performed using robust methods, such as nested 10-fold cross-validation and standardized IBSI methods for feature selection, which provide greater reliability than the non-external model approach presented by Yardimci A.H. et al. and ensure greater reproducibility of results.

Shin et al. [20] assessed MRI radiomics for predicting pCR post-neoadjuvant chemoradiotherapy in locally advanced rectal cancer, underscoring the superiority of model performance over experienced radiologists. Their findings, which revealed an AUC of 0.82 for the T2-weighted and merged models, align closely in accuracy with our best model performance but utilize both pre- and post-treatment imaging data, whereas our approach innovatively leverages pre-treatment MRI alone. This further emphasizes the potential of early prediction frameworks like ours for more proactive treatment planning.

Sun Y. et al. [21] investigated whether a radiomic model based on staging T2-weighted MR images was able to identify the pathological features of rectal cancer. With their model, an AUC of 0.852 was obtained for identifying the histopathological T stage of the tumor. Ma X. et al. [22] tested the performance of radiomics models from T2-weighted images for the degree of differentiation, T stage, and N stage. Least absolute shrinkage and selection operator were used to select features, and multilayer perceptron (MLP), logistic regression (LR), SVM, decision tree (DT), RF, and kNN were trained using 5-fold cross-validation to build a prediction model. They obtained a good AUC from 0.651 to 0.983. In these two papers, differently from our study, patients did not undergo neoadjuvant therapy but direct surgery after the staging, and the examinations were performed with a 3T scanner. This is a limitation for the clinical application of these models, as pCRT is now the standard of care for the treatment of rectal cancer.

Wen L et al. [23] showed that a model based on radiomic features extracted from T2-weighted images before and after pCRT had a good performance in the detection of pCR that outperformed the evaluation of radiologists.

On the other hand, a study by Crimì F. et al. [24] showed how parameters extracted only from restaging T2-weighted MR images were not able to predict the response to chemoradiation.

Other studies employed a combination of T2-weighted and DWI sequences for pCR prediction in rectal cancer. Cai L. et al. [25], in a recent large retrospective multicentric study, proposed an automated pipeline from tumor segmentation to feature extraction and analysis using T2-weighted and DWI images from staging MRI for the identification of pCR and extramural vascular invasion (EMVI). They obtained a good AUC for EMVI identification (0.62 to 0.76) and moderate AUC for pCR prediction (0.53–0.62), with the best model for pCR combining DWI and T2-weighted features. Compared to our study, Cai L. et al. used an automatic segmentation method (vs. our manual delineation of the tumor) and a larger cohort of patients, with MR images from nine different centers, but while their results for EMVI were promising, their results for pCR were less satisfactory. Peng W. et al. [26] used radiomic parameters derived from staging and restaging MRI using both T2-weighted and DWI images, but they concluded that MRI-based radiomic models exhibited no definite added value compared with the clinical models in the prediction of pCR after pCRT. Santini D. et al. [27] demonstrated that a radiomic model based on T2-weighted and apparent diffusion coefficient (ADC) maps acquired before and after pCRT was able to differentiate good from poor responders.

Wei Q et al. [28], using MRI radiomic features extracted from staging and restaging T2-weighted and DWI images, achieved prediction models for pCR with an AUC up to 0.872.

Some research groups extracted features from T2-weighted, contrast-enhanced T1-weighted, and DWI images to build their radiomic predictive model. El Homsi M. et al. [29] performed a dual-center retrospective study to assess the role of pre-treatment MRI radiomics features in predicting pCR of rectal cancer after pCRT. They manually delineated T1-weighted before and after contrast injection, T2-weighted, and DWI sequences, extracting radiomics features to elaborate different models of prediction. They achieved for the models AUCs ranging from 0.7 to 0.9, with a high b-value DWI model demonstrating the best AUC. Shi L. et al. [30] similarly used T2-weighted, diffusion-weighted, and dynamic contrast-enhanced MRI features extracted from staging MRI scans before pCRT for pCR prediction, obtaining an AUC of 0.84.

In other recent studies [31,32], MRI models based on both MRI and clinical features showed good accuracy in the detection of pCR.

There were also some researches focusing on a combination of MRI and PET radiomic features for the prediction of complete response after pCRT, achieving good results [33,34].

To summarize, radiomic models for predicting pCR after neoadjuvant therapy in rectal cancer from MR images show mixed results. Models using T2-weighted images have demonstrated moderate-to-high potential for pCR prediction, with some achieving AUCs above 0.8, but the results can vary significantly depending on image type (pre-treatment vs. post-treatment), methods, and whether additional imaging sequences are included.

When multiple image modalities were combined, the accuracy tended to improve, though not always substantially compared to single-modality models. Automated segmentation approaches and multicentric datasets offered broader applicability but sometimes showed lower performances compared to more targeted studies.

Our study differs from all the others for radiomic models aimed at pCR prediction because it is based solely on pre-treatment T2-weighted images (high-resolution axial oblique images).

Some limitations of our study should be acknowledged. First of all, it was a pilot monocentric study with a limited patient cohort, limiting how broadly the results can apply to other settings. However, the study had a prospective enrollment of patients, and our aim for the near future is to involve other centers in the study for a new model training with a larger patient cohort and greater external validation, also allowing further statistical analyses of the results on sub-cohorts of patients subdivided according to other clinical variables. We believe that the potential of a model based on the one presented in this study, enriched with clinical data and validated and tested in a larger cohort of patients, can be extremely useful in daily clinical practice. The goal is to better inform the patient and the multidisciplinary team about the therapeutic options, with the aim of improving patient care and moving towards rectal-sparing treatment.

Another limitation lies in the use of a manual segmentation procedure to identify the VOIs, which, even if performed by a radiologist with decades of experience in rectal cancer diagnostics, can introduce variability depending on the observer. In the future, we intend to conduct an analysis aimed at identifying radiomic features stable across segment variation and, parallelly, to evaluate the effect of an automatic segmentation on the model performance.

The use of only one sequence (T2-weighted images) for the radiomics analysis is another limitation, and the use of multiple sequences could have improved the model’s performance. For the current plot study, we decided to use only high-resolution images where the tumor is better appreciable to ease the manual segmentation procedure for the radiologists. In the future, we intend to include other MR sequences to verify the possible improvement of the model.

It should be noted that our current training and external validation performances are not suitable for clinical practice, but, as already stated, this is a pilot study aimed at evaluating the potentiality of applying MRI radiomics analysis to rectal cancer treatment response prediction.

## 5. Conclusions

Our study demonstrates the potential of radiomic-based machine learning models for the prediction of pCR in rectal cancer after pCRT starting from staging MR images. These models showed promising discriminative capabilities, emphasizing the importance of integrating advanced imaging and computational methodologies in the pursuit of personalized rectal cancer management. Our results underscore the potential of radiomic features in the prediction of response to pCRT from the initial staging of the patients with locally advanced rectal cancer.

## Figures and Tables

**Table 1:** (**A**) Model of 3 ensembles of random forest classifiers|Classification performance in terms of AUC, accuracy, sensitivity, specificity, PPV, and NPV, corresponding 95% confidence interval, and statistical significance with respect to chance/random classification (*p*-value). Performances are reported for training, validation, and internal-testing. (**B**) Model of 3 ensembles of support vector machine classifiers|Classification performance in terms of AUC, accuracy, sensitivity, specificity, PPV, and NPV, corresponding 95% confidence interval, and statistical significance with respect to chance/random classification (*p*-value). Performances are reported for training, validation, and internal-testing. (**C**) Model of 3 ensembles of k-nearest neighbor classifiers|Classification performance in terms of AUC, accuracy, sensitivity, specificity, PPV, and NPV, corresponding 95% confidence interval, and statistical significance with respect to chance/random classification (*p*-value). Performances are reported for training, validation, and internal-testing.

(**A**)				
	**Training**	**Validation**	**Internal Testing (Mean)**	**Internal Testing (Majority Vote—50% Threshold)**
ROC-AUC (%) (95% CI)	100 * (9–100)	74 ** (72–77)	73 ** (65–81)	73
Accuracy (%) (95% CI)	100 * (99–100)	71 ** (66–76)	70 ** (61–79)	72
Sensitivity (%) (95% CI]	100 * (99–100)	78 ** (73–84)	78 ** (73–84)	81
Specificity (%) (95% CI]	100 * (99–100)	52 ** (46–58)	48 ** (29–67)	50
PPV (%) (95% CI)	100 * (99–100)	82 ** (79–86)	80 ** (73–87)	81
NPV (%) (95% CI)	100 * (99–100)	52 ** (45–58)	46 ** (31–61)	50
(**B**)				
	**Training**	**Validation**	**Internal Testing (Mean)**	**Internal Testing (Majority Vote—50% Threshold)**
ROC-AUC (%) (95% CI)	76 ** (76–77)	74 ** (70–78)	69 ** (66–73)	70
Accuracy (%) (95% CI)	71 ** (70–71)	68 ** (67–68)	63 ** (55–72)	67
Sensitivity (%) (95% CI)	68 ** (67–69)	66 ** (65–68)	62 ** (55–69)	63
Specificity (%) (95% CI)	73 ** (72–75)	71 ** (68–73)	67 (38–95)	75
PPV (%) (95% CI)	73 ** (72–73)	86 ** (82–90)	83 * (71–96)	87
NPV (%) (95% CI)	69 ** (68–70)	49 ** (46–51)	40 ** (30–50)	44
(**C**)				
	**Training**	**Validation**	**Internal Testing (Mean)**	**Internal Testing (Majority Vote—50% Threshold)**
ROC-AUC (%) (95% CI)	90 ** (89–90)	51 ** (46–55)	51 ** (40–63)	51
Accuracy (%) (95% CI)	80 ** (79–81)	52 ** (48–57)	54 ** (50–58)	56
Sensitivity (%) (95% CI)	70 ** (70–71)	56 ** (51–60)	55 ** (52–58)	58
Specificity (%) (95% CI)	90 ** (88–92)	44 ** (35–54)	50 ** (38–62)	50
PPV (%) (95% CI)	88 ** (86–90)	72 ** (67–77)	74 ** (69–79)	75
NPV (%) (95% CI)	75 ** (75–75)	29 ** (23–35)	30 ** (25–35)	31

* *p*-value < 0.05/** *p*-value < 0.005.

**Table 2:** Ensemble of random forest classifiers. The 6 predictors are sorted in descending order according to their statistical significance and relevance.

#	Feature Family	Feature Nomenclature	Median in the pCRT Not Responding Class (95% CI)	Median in the pCR Class (95% CI)	Uncorrected *p*-Value	Corrected *p*-Value
1	Neighbouring Grey Level Dependence Matrix	MR-T2W_LoG_high Dependence High Grey Level Emphasis	7120.79 [5690.59–8550.98]	4117.66 [2431.26–5804.06]	<0.005	<0.05
2	Morphology	MR-T2W_least Axis Length	24.48 [21.68–27.28]	17.27 [14.56–19.99]	<0.05	<0.05
3	Intensity-Based Statistics	MR-T2W_square_10th Percentile	30.26 [22.1–38.42]	48.43 [23.34–73.52]	<0.05	0.24
4	Grey-Level Co-Occurrence Matrix	MR-T2W_exponential_difference Variance	36.44 [28.61–44.26]	43.76 [26.81–60.72]	5.21 × 10^−2^	0.31
5	Grey-Level Co-Occurrence Matrix	MR-T2W_logarithm_difference Entropy	4.41 [4.28–4.55]	4.68 [4.53–4.82]	0.15	0.88
6	Neighbouring Grey Level Dependence Matrix	MR-T2W_LoG_low Dependence High Grey Level Emphasis	779.14 [705.33–852.94]	924.49 [803.63–1045.35]	0.3	1

## Data Availability

Data are available upon reasonable request from corresponding author.
